# Evaluation of prevention behaviour and its influencing factors with respect to cancer screening

**DOI:** 10.1007/s00432-022-03963-w

**Published:** 2022-03-07

**Authors:** Adam Dawid, Christoph Borzikowsky, Sandra Freitag-Wolf, Sabine Herlitzius, Hans-Jürgen Wenz, Jörg Wiltfang, Katrin Hertrampf

**Affiliations:** 1 Department of Oral and Maxillofacial Surgery, University Hospital Schleswig-Holstein, Campus Kiel, Arnold-Heller-Str. 3, Building B, 24105 Kiel, Germany; 2 Institute of Medical Informatics and Statistics, Kiel University, University Hospital Schleswig-Holstein, Campus Kiel, Fleckenstr. 6, 24105 Kiel, Germany; 3 Occupational Medicine, City of Kiel Department of Health, Fleethörn 18, 24103 Kiel, Germany; 4 Department of Prosthodontics, Propaedeutics and Dental Materials, University Hospital Schleswig-Holstein, Campus Kiel, Arnold-Heller-Str. 3, Building B, 24105 Kiel, Germany

**Keywords:** Early detection, Prevention, Cancer, Barriers, Questionnaire, Cancer screening

## Abstract

**Purpose:**

Every year, about 4.6 million people are diagnosed with cancer in Europe. However, based on preclinical changes and using appropriate examination procedures certain cancers can be detected in symptom-free patients at an early stage and treatment initiated.

In Germany, various cancer screening examinations are currently offered to the relevant age groups and sexes free of charge. Participation rates are affected by a number of factors and barriers. The study aimed at identifying potential obstacles and barriers to uptake, taking into account demographic and socio-economic variables.

**Materials and methods:**

Data collection was conducted in the context of routine examination appointments at the City of Kiel Occupational Health Department from September 2013 to September 2014 using an anonymised questionnaire. In addition to recording socio-demographic data and tobacco consumption, the questionnaire also catalogued participation in statutory health insurance cancer screening examinations using the “stages of change” from the Transtheoretical Model. Eight potential barriers to participation were recorded.

**Results:**

The results are based on 718 completed questionnaires. It was found that women, older age, and non-smoking status were associated with a higher probability of participating in cancer screening. It was also found that various barriers affecting (regular) participation were perceived significantly different according to the individual stages of change. This influence of the stages was moderated by gender.

**Conclusion:**

The results showed interesting trends in the different barriers and how they are influenced by socioeconomic factors and the stages of change. Especially the stages require different gender-specific approaches to mobilisation for cancer screening.

## Introduction

Every year, approximately 4.6 million people are diagnosed with cancer in Europe, with 2.1 million dying from it. The most frequent forms are breast cancer, colorectal cancer, lung cancer and prostate cancer, together accounting for half of all new cancer cases (Ferlay et al. 2018). Germany sits in the middle of the EU. Here, the most frequently diagnosed new cancer cases in men are cancers of the prostate, lung, and bowel, and in women of the mammary gland, bowel, and lung. In 2016, 492,000 people were newly diagnosed with cancer in Germany, while 230,000 people succumbed to their cancers. Thus, cancer is the second most common cause of death in Germany after cardiovascular diseases, accounting for 25.3% of deaths (Robert Koch Institut 2019).

The World Health Organization estimates that more than 30–50% of all cancers worldwide could be prevented simply by reducing exposure to lifestyle-associated risk factors such as smoking, alcohol, diet, obesity, and physical inactivity, and implementing evidence-based prevention strategies (WHO 2018). Certain cancers can be detected at an early stage based on preclinical changes in symptom-free patients using appropriate examination procedures, and treatment can be initiated. This early-detection approach aims to improve the chances of a cure and thus survival via early treatment (Hense 2018; Robert Koch Institut 2016; Starker et al. 2018).

In 1971, the first early cancer-detection programmes were included in the scope of benefits for statutory health insurance in the Federal Republic of Germany. The exact nature of cancer screening examinations is regulated by the early detection of cancer guidelines (Krebsfrüherkennungs-Richtlinie) and focussed on the target groups by age and gender with a high incidence of the respective cancers (Gemeinsamer Bundesausschuss 2020a, b). Currently, examinations for early detection of cancer of the skin, intestine, uterus, breast, and prostate are offered to the respective age groups and genders that have a high incidence of respective cancer. Participation is voluntary (Gemeinsame Bundesausschuss 2020a, b; Starker et al. 2018).

Despite access free of charge for all target groups with statutory insurance, the offer is not taken up by all those eligible within the age groups. For example, according to the German GEDA 2014/2015-EHIS survey, the take-up rate for cervical cancer screening in Germany in the preceding 3 years was 80.4%, above the European average of 70.8%. For mammography, Germany also ranks above the European average of 68.7% with 74.2% uptake. In the test for occult blood in stool, Germany sits in the top three with 50.9% adherence, far above the European average of 31.3%. For colonoscopies, 58.5% of Germans reported having had one within the last 10 years (Robert Koch Institut 2017; Starker et al. 2018). A total of 24.1% of men took part in prostate cancer screening in 2018, with 16.1% of men and 18.1% of women attending a screening for skin cancer (Zentralinstitut für die kassenärztliche Versorgung 2021).

Participation rates for cancer screening are influenced not only by the form of examination but also by numerous other factors and barriers. With regard to the influence of gender, women showed higher rates of participation than men (Robert Koch Institut 2016; Scheffer et al. 2006; Sieverding 2011). Furthermore, willingness to participate increased with the level of professional qualification, especially among women ((Bergmann et al. 2005; Robert Koch Institut, 2016; Scheffer et al. 2006).

The level of information about screening examinations also had a significant positive effect on their uptake (Bergmann et al. 2005; Jia et al. 2013; Miri et al. 2018; Saei Ghare Naz et al. 2018). In addition to greater motivation, well-informed individuals have an increased awareness of the benefits of cancer screening. They also have a lesser perception of the barriers to a cancer screening examination (Jia et al. 2013; Miri et al. 2018; Tavafian et al. 2009; Veena et al. 2015). A comprehensive review showed however that information transfer alone was not sufficient to initiate motivation to change (Contento et al. 1995).

An important aspect of increasing accessibility to cancer screening is raising awareness among specific target groups. The extent to which the perception of prevention opportunities differs or otherwise among members of certain professional groups with similar socio-economic backgrounds or similar behaviour towards risk factors has not yet been sufficiently investigated.

The aim of this paper was therefore to identify reasons (1) why cancer screening measures are taken advantage of and (2) what possible obstacles and barriers to uptake may be, taking into account demographic and socioeconomic variables.

## Materials and methods

### Study design

The study was conducted as single-centre observational study with one measurement point (cross-sectional study).

The study population consisted of individuals who presented to the City of Kiel Department of Health, Occupational Medicine for a routine occupational health examination appointment. These individuals from the following occupational groups are required to attend consultations for various purposes including statutory occupational health, return to work and initial examinations.

### Inclusion and exclusion criteria

Inclusion criteria were a fixed appointment with the occupational health service, a minimum age of 16 years (being of legal age), and the exercise of one of the following professions: opera house staff (orchestra, chorus singers, stage craftsmen, make-up artists, painters, carpenters), real estate industry staff (locksmiths, caretakers, cleaners, civil engineering, sewage works workers, city drainage), Waste disposal Kiel staff (waste disposal workers, street cleaning), professional fire brigade staff (administration office staff), paramedics, employees of the public health department, parks department staff (landscape gardeners, cemetery gardeners, foresters), seaport staff (harbour workers, security personnel), and members of the voluntary fire brigade (FFW).

Members of other occupational groups not listed, those who had not yet reached the age of 16 and those without a declaration of consent were excluded from the survey.

### Study procedure

The interview took place in the context of the pre-arranged appointments. Subjects who fulfilled the inclusion criteria received the patient information and were asked to participate in the study. The questionnaire was filled out by participating subjects in the waiting area of the occupational medicine department. Subjects who did not want to participate in the study were documented on a separate list. The Ethical Committee of the Medical Faculty of the University of Kiel (AZ D476/13) approved the study.

### Survey instrument

Data collection was anonymised by means of a questionnaire. In addition to socio-demographic information on age, gender, and occupation, questions were also asked regarding tobacco and alcohol consumption as well as regarding reasons for and dates of participants’ most recent interaction with a doctor. The questionnaire, based on Hartwig and Waller (Hartwig and Waller 2006), additionally recorded participation in cancer screening examinations under statutory health insurance using the “stages of change” (SOC) from the Transtheoretical Model of Health Behaviour (Prochaska and DiClemente 2005). This is a concept for describing, explaining, predicting, and influencing intentional changes in behaviour. It is based on the assumption that decision-making processes regarding change (risk) behaviour progress through several qualitatively different and successive stages of behaviour change, the so-called “stages of change”. Individuals in the respective stages differ from one other in terms of the advantages and disadvantages they perceive from behavioural change. The Transtheoretical Model provides the empirical background for developing recommendations for action within a patient- or needs-oriented intervention with a focus on stage-specific requirements, strategies, and goals (Jong-Meyer de and Engberding 1996; Keller 2004).

The stages of change are as follows:

1. Precontemplation: people at this stage have no intention of changing a problematic behaviour in the foreseeable future. They are not aware of their (mis)behaviour or do not see it as problematic.
2. Contemplation: these people are aware of their problem. They are characterised by a certain ambivalence towards their problematic behaviour and weigh up the pros and cons of potentially changing their behaviour.
3. Preparation: these people concretely plan to change their problematic behaviour and take the first steps towards a change in behaviour.
4. Action: individuals in this stage make an obvious visible behavioural change in the direction of the desired behaviour. However, as the new behaviour pattern has yet to be habituated, there is still a risk of relapse into old behaviour patterns.
5. Maintenance: these people have long given up their problematic behaviour and accordingly have a decreasing risk of falling back into their old pattern of behaviour.

The corresponding question in the survey regarding the individual stages was: “Have you ever taken part in cancer screening?” The following six response options were available: “No, and I’m not concerned about it yet either” (= precontemplation), “No, and I probably won’t do in future either” (= precontemplation). This subdivision of the precontemplation stage was a result of preliminary studies within the research project by Hartwig and Waller (2006) having already shown that a portion of the population simply rejects cancer screening outright (Hartwig and Waller 2006).

Other answer options were: “No, but I’ve thought about it” (= contemplation), “No, but I intend to soon” (= preparation), “Yes, once” (= action), and “Yes, several times” (= maintenance).

### Measuring the barriers to early cancer detection

Following Hartwig and Waller, additional statements were also formulated for ranking potential circumstances or situations that may have a possible influence on participation in cancer screening examinations:

1. You have to make extra appointments/be prepared to accept waiting times.
2. No one particularly tells you about cancer screening.
3. I do not know what cancer screening’s about.
4. I find having these kinds of examinations unpleasant.
5. Friends/acquaintances my age do not go for cancer screening either.
6. I do not have much time for these kinds of examinations.
7. Potentially receiving a positive result is a deterrent.
8. It is difficult to find a suitable doctor.

Subjects evaluated these eight hurdles using a four-point Likert scale from 1 = “strongly disagree” to 4 = “strongly agree”, with the additional option of “don’t know”.

For better comparability, the mean evaluations for the eight individual circumstances were calculated by giving the rating “strong disagreement” a value of 1, “disagreement” a value of 2, “agreement” a value of 3 and “strong agreement” a value of 4. Thus, each mean value corresponds to the average extent of the perceived obstacle.

The aim of these questions was to find out which barriers have the most influence on participation behaviour in general, as well as within the different stages of change.

### Statistical evaluation

Statistical evaluation of the questionnaire was primarily descriptive (means and standard deviations for continuous variables, or absolute and relative frequencies for categorical variables).

The eight statements on potential circumstances or situations that might influence participation (barriers to early cancer detection) were rated ordinally using a four-point Likert scale. The Kruskal–Wallis test, a non-parametric statistical test, was used to identify differences between the study participants from the various stages of change. The Kruskal–Wallis test assesses whether groups differ significantly from one other with respect to their responses to the individual obstacles. The Kruskal–Wallis test was also stratified according to gender and smoking behaviour to check the robustness of the empirical findings (subgroup analyses). Group comparisons regarding categorical variables (e.g. women vs men for gender) were performed using the Fischer exact test. *P* values < 0.05 were designated as statistically significant. All analyses were carried out using the IBM SPSS Statistics 25 package.

## Results

### Participants

In the survey period from September 2013 to September 2014, a total of 742 individuals fulfilled the inclusion criteria, of whom 718 participated in the study (response rate 96.8%). Men and women participated in the study in equal proportions (49.0% each), while 14 people (2.0%) did not indicate their gender. Participants were aged between 16 and 65 years, with an average age of 38.2 years (SD = 12.9). The majority of participants (*n* = 436, 60.7%) were younger than 45 years. Almost one third of study participants (*n* = 230, 32.0%) were smokers, with a slightly higher (but not significantly so) proportion of smokers among men (*n* = 124, 35.2%) compared to women (*n* = 100, 28.4%, *p* = 0.063). The distribution of the study population is detailed in Table 1. Due to the somewhat questionable information on alcohol consumption, this information is only presented in the table and is not taken into account in further evaluations.

**Table 1:** Distribution of the study population by gender, age group, smoking behaviour, and alcohol consumption

	Total (*n* = 718) (%)	Women (*n* = 352)	Men (*n* = 352)
≤ 44 years	436 (60.7)	219 (62.2)	217 (61.6)
45–54 years	172 (24.0)	87 (24.7)	84 (23.9)
55–65 years	92 (12.8)	46 (13.1)	46 (13.1)
Smokers	230 (32.0)	100 (28.4)	124 (35.2)
Non-smokers	487 (67.8)	251 (71.3)	228 (64.7)
Daily / several times a week alcoholic drinks	68 (9.4)	28 (8.0)	40 (11.3)
Once a week and less alcoholic drinks	520 (72.4)	247 (70.2)	273 (77.6)
No alcohol consumption	106 (14.8)	71 (20.2)	35 (9.9)

14 study participants did not report gender, 18 participants did not report age and one participant did not report smoking behaviour, 10 participants did not report their alcohol consumption

### Uptake of cancer screening measures

Participants were then further asked about their participation in cancer screening examinations and the available response options were assigned to the individual stages of change. Thus, 120 study participants (16.8%) neither engaged in screening examinations nor planned to do so in the future (precontemplation). A further 164 people (22.8%, contemplation) had thought about participating, while 66 people (9.2%) intended to participate in a screening examination in the near future (preparation).

**Fig. 1:**
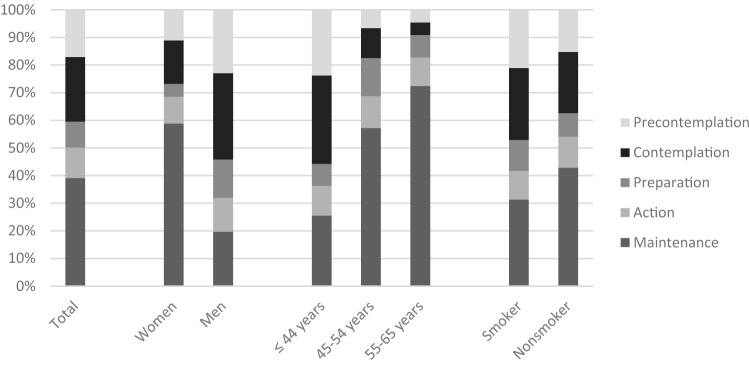
Distribution of study population by stage of change (participation in cancer screening examinations) stratified by sex, age group and occupational category

Of those who had already taken part in cancer screening, 77 people (10.7%) had attended once (action), while 275 (38.3%) had taken part several times (maintenance, Table 2 and Fig. 1).

**Table 2:** Distribution of study population by gender, and participation in cancer screening examinations broken down by stage of change

Participation in cancer screening examinations	Stage	Women (*n* = 352) (%)	Men (*n* = 352) (%)	Total (*n* = 718) (%)
No, and I probably won’t do in future either	Precontemplation	38 (10.8)	79 (22.4)	9 (1.3)
No, and I’m not concerned about it yet either				111 (15.5)
No, but I’ve thought about it	Contemplation	54 (15.3)	108 (30.6)	164 (22.8)
No, but I intend to soon	Preparation	16 (4.5)	48 (13.6)	66 (9.2)
Yes, once	Action	33 (9.4)	42 (11.9)	77 (10.7)
Yes, several times	Maintenance	202 (57.4)	68 (19.3)	275 (38.3)
Missings		9 (2.6)	7 (2.0)	16 (2.2)

16 Study participants did not report their participation in cancer screening examinations

Looking at the genders, a heterogeneous picture emerged. While two-thirds of women (*n* = 235, 66.8%) had already participated in a cancer screening examination (action and maintenance), only *n* = 110 (31.2%, *p* < 0.001) of men had done so. Furthermore, among women *n* = 108 (30.6%) had no experience of cancer screening (precontemplation, contemplation and preparation), while among men the proportion was two-thirds (*n* = 235, 66.6%, *p* < 0.001; Table 2 and Fig. 1).

With increasing age, both willingness to address the issue increased (lack of intention in *n* = 102, 23.4% in the age group < 45 years vs *n* = 4, 4.3% in the age group > 54 years, *p* < 0.001) as well as the likelihood of attending a cancer screening examination (*n* = 157, 36.1% in the age group < 45 years vs *n* = 72, 78.3% in the age group > 54 years, *p* < 0.001; Fig. 1).

Uptake of cancer screening examinations was significantly higher among non-smokers (*n* = 259, 54.1%) than smokers (*n* = 93, 41.7%, *p* = 0.004; Fig. 1).

### Barriers to early cancer detection

The statements attracting the most (Wilson et al. 1998) agreement were: “No one particularly tells you about cancer screening” (*n* = 359, 50.0%), and “Potentially receiving a positive result is a deterrent” (*n* = 301, 41.9%). About two-thirds of study participants (*n* = 478, 66.1%) (strongly) rejected the statement, “I don’t know what cancer screening is all about”. The majority also (strongly) rejected the options, “I don’t have much time for these kinds of examinations” (*n* = 408, 56.8%), “I find having these kinds of examinations unpleasant” (*n* = 394, 54.9%), and “It’s difficult to find a suitable doctor” (*n* = 391, 54.5%).

The barriers with the highest average ratings were: “No one particularly tells you about cancer screening” (*M* = 2.57, SD = 0.84) and “You have to make extra appointments/be prepared to accept waiting times” (*M* = 2.56, SD = 0.84). The obstacle with the lowest average score was, “I don’t know what cancer screening is all about” (*M* = 1.97, SD = 0.87; Table 2).

When the perceived barriers were stratified by study participant behaviour regarding cancer screening (that is, by stage of change), a significantly different response pattern was found for the second barrier: “No one particularly tells you about cancer screening” (*p* < 0.001), third barrier: “I don’t know what cancer screening is all about” (*p* < 0.001), fifth barrier: “Friends/acquaintances my age don’t go for cancer screening either” (*p* < 0.001), sixth barrier: “I don’t have much time for such screening” (*p* < 0.001), and seventh barrier: “Potentially receiving a positive result is a deterrent” (*p* = 0.026; Table 3).

**Table 3:** Distribution of mean values (standard deviations) for perceived barriers to participation in cancer screening across the stages of change

Barriers	Total (*n* = 718)	Precontemplation (*n* = 120)	Contemplation (*n* = 164)	Preparation (*n* = 66)	Action (*n* = 77)	Maintenance (*n* = 275)	*p* Value*
1. You have to make extra appointments/be prepared to accept waiting times	2.56 (0.84)	2.53 (0.86)	2.53 (0.68)	2.53 (0.79)	2.69 (0.82)	2.58 (0.93)	0.551
2. No one particularly tells you about cancer screening	2.57 (0.84)	2.77 (0.79)	2.73 (0.76)	2.55 (0.73)	2.56 (0.84)	2.39 (0.91)	** < 0.001**
3. I do not know what cancer screening’s about	1.97 (0.87)	2.35 (0.87)	2.19 (0.88)	1.92 (0.86)	1.93 (0.87)	1.69 (0.78)	** < 0.001**
4. I find having these kinds of examinations unpleasant	2.18 (0.85)	2.26 (0.93)	2.17 (0.85)	2.41 (0.82)	2.08 (0.85)	2.13 (0.83)	0.136
5. Friends/acquaintances my age do not go for cancer screening either	2.25 (0.94)	2.89 (0.98)	2.52 (0.89)	2.31 (0.87)	2.04 (0.87)	1.86 (0.80)	** < 0.001**
6. I do not have much time for these kinds of examinations	2.28 (0.84)	2.47 (0.87)	2.42 (0.85)	2.36 (0.71)	2.26 (0.78)	2.09 (0.84)	** < 0.001**
7. Potentially receiving a positive result is a deterrent	2.40 (0.97)	2.42 (1.02)	2.55 (0.97)	2.51 (0.92)	2.50 (0.93)	2.25 (0.96)	**0.026**
8. It’s difficult to find a suitable doctor	2.22 (0.82)	2.27 (0.90)	2.38 (0.81)	2.15 (0.69)	2.16 (0.85)	2.14 (0.82)	0.125

^*^Kruskal–Wallis test regarding the stages of change, *p *values < 0.05 significant group differences (highlighted in bold)

When comparing the genders, we observed a significantly different response pattern between the stages for the second obstacle: “No one particularly tells you about cancer screening” (*p* = 0.008), third obstacle: “I don’t know what cancer screening is all about” (*p *< 0.001), and fifth obstacle: “Friends/acquaintances my age don’t go for cancer screening either” (*p* < 0.001). In contrast to the initial evaluation with no stratification by gender, there was no significantly different response pattern between the stages for the sixth obstacle: “I don’t have much time for these kinds of examinations” (*p* = 0.064), and the seventh obstacle: “Potentially receiving a positive result is a deterrent” (*p* = 0.185).

For men, a significantly different response pattern emerged between the stages for the third barrier: “I don’t know what cancer screening is all about” (*p* < 0.001), fifth barrier: “Friends/acquaintances my age don’t go for cancer screening either” (*p* < 0.001), and sixth barrier: “I don’t have much time for these kinds of examinations” (*p* = 0.002). In contrast to the earlier evaluation with no stratification by gender, no significantly different response pattern was observed between the stages for the second obstacle: “No one particularly tells you about cancer screening” (*p* = 0.094), and the seventh obstacle: “Potentially receiving a positive result is a deterrent” (*p* = 0.740).

## Discussion

This study used a questionnaire to investigate possible factors that can positively or negatively influence the decision to participate in a cancer screening examination. Our study shows that age, gender, and the risk factor tobacco consumption had an influence on willingness to participate. With regard to gender, it was observed that women (regularly) attended a cancer screening examination far more often than men (66.8 vs 31.2%).

A similar trend can be seen in the 2008–2011 Robert Koch Institute (RKI) study on the health of adults 18 to 79 years old in Germany (DEGS1). Here, 67.2% of women (20 years and older) regularly participated in cancer screening examinations, but only 40.0% of men (35 years and older). However, participation rates in gender-independent cancer screening examinations differ less according to the same study (Robert Koch Institut, 2016). Data from the Central Institute for Statutory Health Insurance Physicians (Zi), based on billing data for statutory health insurance-accredited medical care, also shows that participation in gender-independent cancer screening examinations was slightly higher among women than among men (Starker et al. 2018).

In this present study, we also observed that experience of cancer screening examinations and regular participation in them increased with age. The same trend was identified in DEGS1 (2008–2011), too, although regular participation fell again among women from the age of 70 (Robert Koch Institut, 2016). Similarly, both GEDA 2014/2015-EHIS and the Zi billing data report participation figures increasing with age until around the age of 70 (Robert Koch Institut, 2016; Starker et al. 2018).

However, not only are cancer screening offerings differentiated according to gender but they are offered to men at a later age than women, something which may influence our cohort’s differing reported perceptions of these examinations. Interpretation of the data must also take into account the fact that there is currently no offering for one of the age groups queried about participation.

With regard to the risk factor “tobacco consumption”, almost one-third of our cohort reported being smokers. By comparison, the RKI’s most recent survey (GEDA 2014/2015-EHIS) identified 20.8% of women and 27.0% of men in Germany as smoking at least occasionally, a lower proportion than in our cohort (Zeiher et al. 2017). In our study, refusal to undergo cancer screening examinations was greater—and (regular) participation lower—among smokers than among non-smokers. The study by Sänger (2014), in this case on prostate cancer check-ups, also observed that non-smoking men participated more frequently in screening (Sänger 2014).

Comparing our results with respect to the influence of particular barriers on (regular) participation in cancer screening and their classification within the stages of change, the following may be observed.

When considering the different potential perceived barriers that had an influence in this study on (regular) uptake of cancer screening examinations, the barrier, “No one particularly tells you about cancer screening” was seen as the greatest. Hartwig and Waller (2006) also observed the same tendency in their study among men aged 45–60 years using the same set of questions regarding barriers (Hartwig and Waller 2006). Bourdeanu (2020) also observed that a doctor’s recommendation was a predictor for a mammogram in her survey of Lebanese women (Bourdeanu et al. 2020).

Similarly, the Robert Koch Institute described one possible reason for the routinely high participation of women between 50 and 69 years of age in cancer screening examinations as being biennial written invitations to the mammography screening programme, an initiative which actively targets the obstacle of non-referral (Robert Koch Institut, 2016).

The least important perceived barrier in this study, “I don’t know what cancer screening is about”, was also described by Hartwig and Waller (2006) as the least important (Hartwig and Waller 2006). These findings do, however, contrast to some degree with the “Gesundheit in Deutschland aktuell 2010” [Health in Germany, actual 2010] (GEDA 2010) study, in which the need for better information for eligible individuals was highlighted by respondents as a reason for non-participation (Robert Koch Institut, 2016). Internationally, a lack of knowledge about cancer screening examinations is also a frequently described risk factor for non-participation. In their systematic review on breast and cervical cancer, for example, Ackerson and Preston (2009) found that lack of adherence to cancer screening examinations often affects women who do not know about them or do not seek them out (Ackerson and Preston 2009). Similarly, Gebru and Gerbaba (2016) (via in-depth interviews with Ethiopian women), McFarland et al. (2016) (via an integrated review in sub-Saharan Africa), and Parajuli et al. (2020) (via interviews with Bhutanese refugee women) all reported that lack of knowledge was the biggest barrier to cervical cancer screening (Gebru and Gerbaba 2016; McFarland et al. 2016; Parajuli et al. 2020). Veena et al. (2015), using a cross-sectional study of women aged 40–65 in India, likewise described a lack of knowledge as the biggest barrier to participation in breast cancer screening (Veena et al. 2015).

As has previously been described, an additional strongly influencing factor is the likelihood of participation being proportional to an individual’s assumptions about the screening behaviour of other people their age, something which seems to consolidate in their inaction especially those individuals who do not attend cancer screening examinations (Sieverding 2011).

The fact that certain barriers and obstacles to cancer screening uptake are perceived differently within the individual stages of change has been described internationally with respect to a number of different procedures. The perceived barriers to breast self-examination, for example, were smaller among women who had already performed breast self-examination (Tavafian et al. 2009). With respect to mammography, women at the no-intention level perceived greater barriers compared to women who classified themselves at the intention level (Lee-Lin et al. 2016; Salinas-Martinez et al. 2018).

Women had lower perceived barrier scores for Pap smears within the stages of action and maintenance (Tung et al. 2017), such that perceived barriers to Pap smear cervical cancer screening were found to be a predictor of each stage of change (Miri et al. 2018). By contrast, a Nepali study found that education level, but not perceived barriers, was significantly associated with participation in cervical cancer screening (Acharya and Karmacharya 2017). Similarly, study participants were more likely to adhere to colonoscopy screening guidelines if they perceived fewer barriers (Williams et al. 2018).

### Strengths and weaknesses

The strength of the study is reflected in the very high response rate, which thus represents a good cross-section of the selected cohort. Nevertheless, the study also has ā weakness that are explained below and should be taken into account when interpreting the results. The survey was a single centre with subjects from one office only. When interpreting the results, it must be taken into account that no complete data sets were available for age and gender. However, the missing values were within a reasonable range. Nevertheless, this should be kept in mind when interpreting the results. With regard to smoking behaviour, occasional smokers with light smoking behaviour and smokers with heavy smoking behaviour were all included in the smokers group. This could lead to an overinterpretation of smoking as a risk factor.

## Conclusion

In summary, our study found that women were more likely to (regularly) engage with cancer screening measures than men. Those of greater age also had a higher probability of (regular) participation than the younger. In addition, non-smokers were more likely to (regularly) attend cancer screening examinations than smokers.

Furthermore, it was found that different barriers were perceived differently in the different stages of change, but the influence of the stages was moderated by gender. Thus, it is advisable to consider not only the stages but also the interaction of the stages and gender when developing concepts to overcome the hurdles.

## Data Availability

Not applicable.
